# Testing and Diagnosis of Clostridioides difficile Infection in Special Scenarios: A Systematic Review

**DOI:** 10.7759/cureus.59016

**Published:** 2024-04-25

**Authors:** Karan B Singh, Anas Khouri, Deepak Singh, Jose Prieto, Priyata Dutta, Maduka C Nnadozie, Clista Clanton, Esther Morrison, William Sonnier

**Affiliations:** 1 Internal Medicine, Frederick P. Whiddon College of Medicine at the University of South Alabama, Mobile, USA; 2 Internal Medicine, Caribbean Medical University, Willemstad, CUW; 3 Internal Medicine, Loyola University MacNeal Hospital, Berwyn, USA; 4 Internal Medicine, Trinity Health St. Joseph Mercy Ann Arbor, Ann Arbor, USA; 5 Internal Medicine, AtlantiCare Regional Medical Center, Atlantic City, USA; 6 Biomedical Research, Frederick P. Whiddon College of Medicine at the University of South Alabama, Mobile, USA; 7 Infectious Diseases, Frederick P. Whiddon College of Medicine at the University of South Alabama, Mobile, USA; 8 Gastroenterology and Hepatology, Frederick P. Whiddon College of Medicine at the University of South Alabama, Mobile, USA

**Keywords:** specificity, microbial sensitivity test, infection prevention and control, antibiotic, molecular diagnosis for infectious diseases, toxic megacolon, inflammatory bowel disease, infectious colitis, clostridioides difficile infection

## Abstract

Introduction: *Clostridioides difficile *infection (CDI) is a clinical and laboratory diagnosis. Populations at higher risk of developing disease require a high clinical index of suspicion for laboratory testing to avoid incorrect assumptions of colonization. Common risk factors include recent antibiotic use, elderly (>65 years old), and immunocompromised patients. *C. difficile a*ssays should be ordered in an algorithm approach to diagnose an infection rather than colonization. Screening tests are widely available in hospital systems, but novel molecular testing may aid in diagnosis in patients with inconclusive or discordant antigen and toxin test results.

Methods: Data was extracted from PubMed, Scopus, and Cumulative Index of Nursing and Allied Health Literature (CINAHL) databases based on the keywords “*clostridioides difficile*”, “toxin assay”, and “toxic megacolon”. The data extracted is based on the Preferred Reporting Items for Systematic Reviews and Meta-Analyses (PRISMA) 2020 guidelines. A total of 27 reports were included in this systematic review.

Results: Testing patients with a significant gastrointestinal surgical history, hypogammaglobulinemia, inflammatory bowel disease, intensive care unit, and immunocompromised patients for CDI is highly recommended. Diarrhea in these subsets of patients requires correlation of clinical context and an understanding of assay results to avoid over- and under-treating.

Conclusion: CDI should be considered in all patients with traditional risk factors. Heightened clinical suspicion of CDI is required in patients with hypogammaglobulinemia, transplant recipients, patients with gastrointestinal surgical history, and inflammatory bowel disease. Testing should be limited to patients with clinical manifestations of CDI to ensure a high pretest probability for test interpretation. Healthcare workers should adhere to testing algorithms to optimize yield in the appropriate clinical context. Diagnostic assays should follow a sequential, stepwise approach to categorize the toxin expression status of the bacteria accurately.

## Introduction and background

*Clostridioides difficile* (formerly known as *Clostridium difficile*) is a gram-positive, spore-forming, strictly anaerobic bacillus [1,2]. The organism lives harmoniously in the colon with normal gut flora. This bacterium was first discovered in 1935, and the first case of antibiotic-associated pseudomembranous colitis was subsequently diagnosed in 1978. The strain was originally named *Bacillus difficilis* due to its microscopic appearance and difficult cultivation [3]. This organism is a leading cause of gastrointestinal disease and costs four billion dollars annually [2].

Since the 20th century, the incidence of *C. difficile* infection (CDI) has been increasing worldwide. In 2002, high mortality rates were attributed to a strain called ribotype 027/B1, also known as NAP-1 [3]. Systematic surveillance for CDI prior to 2003 was lacking. After the worldwide outbreak of the NAP-1 strain, in 2011, the Centers for Disease Control and Prevention (CDC) estimated 500,000 CDI cases and 29,000 deaths in the United States [3]. In 2010 the Emergency Infections Program found that 97% of cases were health-care related which is defined as symptoms starting after three days of admission, and 75% of these patients had a history of previous hospitalizations [4]. Trends from another study demonstrated that the incidence of CDI increased from 5.5/10,000 to 11.2/10,000, with more dramatic increases in adults aged > 65 years [4].

The incidence of the NAP-1 variant of *C. difficile* has reached 30% in hospitalized patients, accounting for more than 300,000 newly diagnosed cases per year, and up to 40% of community-acquired infections require hospitalization [3]. Since the discovery of the NAP-1 strain, the number of tests on *C. difficile* has increased [5]. Biochemically, *C. difficile* has two clostridial toxins, enterotoxin (toxin A) and cytotoxin (toxin B). These toxins are encoded by the genes cdtA and cdtB at the pathogenicity locus (PaLoc) [2].

All the *C. difficile* strains can ferment and produce glutamate dehydrogenase (GDH) irrespective of toxigenic properties. This has led to a test for GDH, which has a sensitivity (Sn) ranging from 79.5% to 100%, specificity (Sp) ranging from 82.7% to 100%, and a negative predictive value (NPV) of 100% [6]. GDH testing does not distinguish between toxigenic and nontoxigenic strains; therefore, a confirmatory test is needed for toxin analysis. The best test for detecting toxins is toxigenic culture (TC) due to its high Sn and Sp, but due to its turnaround times, other assays are preferred in the modern era [6-8]. The toxin A/B enzyme immunoassay (EIA) is a common confirmatory test that detects antibodies directed against both virulent clostridial toxins. Sn varies from 53% to 85% and Sp from 91% to 98% [7]. Due to poor Sn, the combination of rapid turn-over tests and a multistep approach is considered to avoid false positives and false negatives [9]. In addition, sole reliance on molecular testing for toxins increases the likelihood of overdiagnosis of *C. difficile*. Therefore, patients with a positive screening test may have asymptomatic colonization or carriage of *C. difficile*, rather than infection. This carriage is common in healthcare-associated facilities and in the community, and it is estimated that the prevalence of this disease ranges from 7% to 18% [10].

This article was previously posted on the Research Square preprint server on February 12, 2024.

## Review

Methods

Design

This systematic review was conducted to establish a comprehensive collection of current data from different databases to align with the most up-to-date evidence-based practice patterns for the workup of CDI. This study followed the Preferred Reporting Items for Systematic Review and Meta-Analysis (PRISMA) checklist. Our research was not registered online.

*Search Strategy and Selection* We evaluated studies that identified the role of biochemical testing of *C. difficile* and the implications of disease severity and the development of toxic megacolon in a subset of patient populations. On February 2, 2023, one investigator (CC) searched PubMed, Scopus, and Cumulative Index of Nursing and Allied Health Literature (CINAHL) databases to identify relevant reports published between 2012 and 2023. The search terms used included index and keyword terms for “*clostridioides difficile*”, “toxin assay”, and “toxic megacolon” (Table 1). The inclusion criteria were English language articles published between 2012 and 2023, with eligibility based on population, type of study, and outcomes. The exclusion criteria were non-English language reports that were experimental (except for one study that was deemed necessary for this review), basic science reports, poor-quality appraisals, pediatric population reports, or outdated guidelines. Six investigators agreed to include and/or exclude the studies based on these criteria before they were finalized in this review. One experimental study was included in this systematic review for the purpose of identifying the hypervirulent strain NAP-1. After removing duplicates, the full-text articles of the search results (n=76) were uploaded to Rayyan, a web-based platform used to organize and manage articles for systematic reviews.

**Table 1:** Key/MeSH words from three different databases search methods MeSH: Medical Subject Headings; CINAHL: Cumulated Index to Nursing and Allied Health Literature

Search engine	Key/MeSH words	Results
PubMed	(("clostridioides difficile"[MeSH Terms] OR "clostridium difficile"[Text Word]) AND "english"[Language] AND (("megacolon, toxic"[MeSH Terms] OR ("megacolon"[All Fields] AND "toxic"[All Fields]) OR "toxic megacolon"[All Fields] OR ("toxic"[All Fields] AND "megacolon"[All Fields])) AND "english"[Language]) AND (("toxin s"[All Fields] OR "toxine"[All Fields] OR "toxins, biological"[MeSH Terms] OR ("toxins"[All Fields] AND "biological"[All Fields]) OR "biological toxins"[All Fields] OR "toxin"[All Fields] OR "toxins"[All Fields]) AND ("analysis"[MeSH Subheading] OR "analysis"[All Fields] OR "assay"[All Fields] OR "biological assay"[MeSH Terms] OR ("biological"[All Fields] AND "assay"[All Fields]) OR "biological assay"[All Fields] OR "assay s"[All Fields] OR "assayed"[All Fields] OR "assaying"[All Fields] OR "assays"[All Fields]) AND "english"[Language])) AND ((english[Filter]) AND (2012:2023[pdat]))	24
Scopus	ALL (clostridioides AND difficile) OR ALL (clostridium AND difficile) AND ALL (toxic AND megacolon) AND ALL (toxin AND assay) AND PUBYEAR > 2011 AND (LIMIT-TO (DOCTYPE , "ar") OR LIMIT-TO (DOCTYPE ,"re")) AND ( LIMIT-TO (LANGUAGE , "English"))	85
CINAHL	(clostridioides difficile or clostridium difficile or c-diff) AND toxic megacolon	37

Data Extraction

Five investigators (AK, JA, MN, PD, DS) extracted five reports each, and the corresponding investigator (KS) extracted three reports from the eligible studies based on the last name of the author, publication year, number of patients, purpose of the study, and results.

Quality Appraisal

Two investigators (KS and AK) independently reviewed each of the 27 included reports for authenticity and quality. We utilized the JBI global website to methodologically assess the transparency of each included report. Review articles (n=18), retrospective cohorts (n=4), guidelines (n=3), experimental studies (n=1), and cross-sectional studies (n=1) were criticized for excellent appraisal. The following instruments were used: Text and Opinion for Review Articles, the revised Appraisal of Guidelines for Research and Evaluation (AGREE) II for guidelines, a diagnostic accuracy tool for cross-sectional studies, an experimental study checklist for experimental studies, and a cohort study checklist for cohort studies.

Results

**Figure 1:**
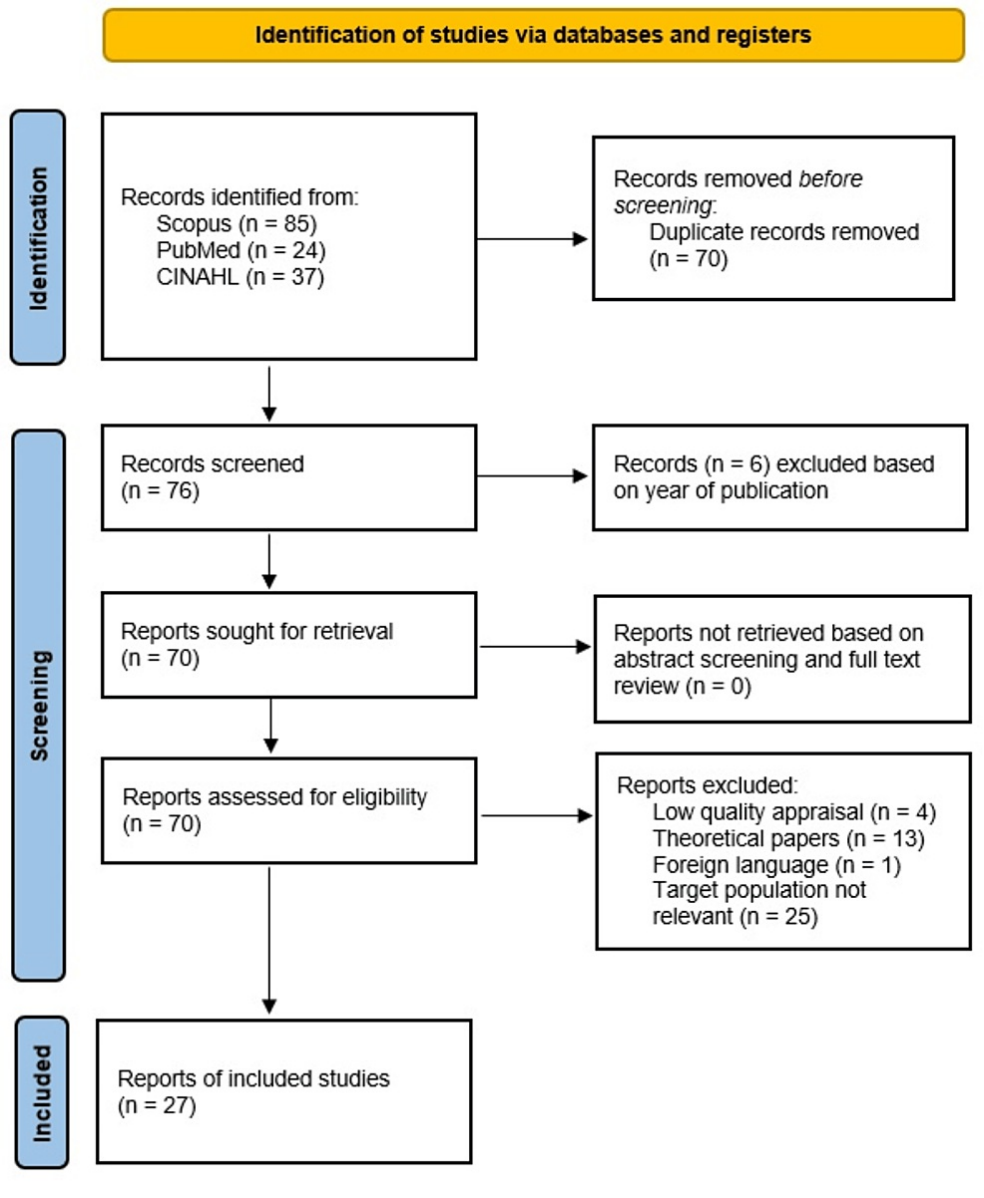
PRISMA 2020 flow diagram for systematic review. PRISMA: Preferred Reporting Items for Systematic Reviews and Meta-Analyses

After identifying 85 records from Scopus, 24 records from PubMed, and 37 records from CINAHL, we removed 70 duplicate records (Figure 1). We manually excluded six records based on publication year. Finally, we excluded records based on abstract screening and not meeting the eligibility criteria. Overall, 27 reports were eligible for inclusion in this systematic review (Table 2) [1-8,10-29].

**Table 2:** Studies included in the systematic review CDI, *Clostridioides difficile *infection; IBD, inflammatory bowel disease; PMC, pseudomembranous colitis; CT, computed tomography; TC, toxigenic culture; GDH EIA, glutamate dehydrogenase enzyme immunoassay; NAAT, nucleic acid amplification test; CCNAs, cytotoxicity neutralization assays; PCR, polymerase chain reaction; CDC, Centers for Disease Control; WBC, white blood cell count; FMT, fecal microbiota transfer; CMV, cytomegalovirus. rCDI, recurrent* Clostridioides difficile* infection; ICU, intensive care unit; CDAD, *Clostridium difficile*-associated diarrhea; ECCO, European Crohn’s and Colitis Organization; AAD, antibiotic-associated diarrhea; TcdB, toxin B gene; PPI, proton pump inhibitors; ESCMID, European Society of Clinical Microbiology and Infectious Diseases

Author, Year	Type of study	Sample (n)	Region	Purpose	Results
Kodadek et al., 2018 [1]	Review article	N/A	United States	Review the prevention and diagnosis of CDI	Diagnosis requires three or more unformed stools in 24 hours or radiographic evidence of ileus or megacolon and a positive laboratory test. Testing should not be repeated within seven days to avoid decreasing specificity.
Martínez-Meléndez et al., 2017 [2]	Review article	N/A	Mexico	Laboratory testing, diagnosis, and prevention of CDI	*Clostridioides difficile* should be tested in Bristol scores of >5 with >3 stool movements. Various testing methods are employed for patients with *C.* *difficile* including TC, GDH EIA, NAAT, CCNAs
Stanley et al., 2013 [3]	Review article	N/A	N/A	*CDI* risk factors, diagnosis, and prevention in specific populations	The primary risk factors include age >65, antibiotic use within the last three months, and hospitalization. Secondary risk factors include hypoalbuminemia, ICU admission, HIV, and autoimmune diseases.
Surawicz et al., 2013 [4]	Guideline	N/A	United States	Clinical guidelines for diagnosis and prevention of rCDI and CDI	The main risk factors for this disease are exposure to antibiotics and the organism. Other risk factors include GI surgery, gastric acid-suppressing medications, IBD, hypogammaglobulinemia, increased age, and poor health.
Lanis et al., 2013 [5]	Experimental study	N/A	United States	*C. difficile* exotoxin, TcdB variant B027 in comparison to other forms of exotoxin variants.	C. *difficile* exotoxin TcdB, especially 027/B1/NAP1 variant is potent and causes symptoms. Both epitomes are potent but B027 is more potent given its resistance genes to antibiotics. These variants also had a brain hemorrhage in mice due to acting on high brain microvascular endothelial.
Xiao et al., 2019 [6]	Review article	N/A	N/A	Review of epidemiology, pathogenesis, and available testing	TC takes three to five days to results and needs complementary testing. GDH has high sensitivity and specificity but needs EIA or NAAT as complimentary testing. NAAT PCR-based testing has high sensitivity, and specificity, but cannot differentiate between asymptomatic colonization and infection.
Abreu Y Abreu et al., 2019 [8]	Guideline	N/A	Mexico	Consensus on the prevention and diagnosis of CDI	TC has the highest sensitivity and specificity followed by CCNAs. A rectal swab with NAAT-PCR can be implemented in patients with ileus or stool samples that are difficult to collect.
Smits et al., 2016 [10]	Review article	N/A	Netherlands, Australia, United Kingdom	Review the diagnosis of CDI	Mainstay diagnosis is clinical suspicion followed by a TC or detection of toxin. Other approaches include antigen and toxin assays followed by nucleic acid testing if there are discordant test results.
Kaiser et al., 2015 [11]	Review article	N/A	United States	Risk factors for CDI in surgical patients	GI surgery, exposure to peri-operative antibiotics, and PPI exposure in surgical patients increase the risk for CDI. In these patients, CDI can lead to recurrent pouchitis in ileo-pouch anal anastomosis and high volume ileostomy output in patients with ileostomy.
Bloukh, 2013 [12]	Review article	N/A	United Arab Emirates	CDI overview of diagnosis and prevention	Diagnosis of CDI is clinical but requires a high clinical suspicion in patients with diarrhea. Multi-step algorithm involving GDH, EIA toxin, and NAAT-PCR is sometimes needed.
Spadao et al., 2014 [13]	Retrospective cohort	2119	Brazil	Diagnosis of CDI in hospitalized hematological patients	The incidence of CDI in this population was 3.1%. The rate of incidence of CDI per 1,000 days of neutropenia varied from 0.78 to 5.45 and per 1,000 patient days varied from 0.78 to 10.24 during the study period. Risk factors associated with death were severe forms of the disease and the use of linezolid.
Gupta et al., 2019 [14]	Retrospective cohort	92	United States	Evaluate toxin testing in escalation of CDI therapy in IBD patients	The toxin-neg PCR+IBD group had a significantly higher rate of antibiotic response and required a lower rate of escalation of therapy compared to only toxin+ patients.
Alrabaa et al., 2013 [15]	Review article	N/A	United States	CDI risk factors, prevention, and diagnosis	Risk factors for CDI include antibiotic exposure, longer length of healthcare stay, higher acuity of care, and advanced age.
Sheitoyan-Pesant et al., 2015 [16]	Retrospective cohort	1527	Canada	Diagnose rCDI within 14-60 days with repeat toxin testing	This type of disease occurs commonly in females and the median age was 73. The probability of developing a first rCDI was 25% and a second rCDI 38% with increasing incidence from 2010-2013.
Rineh et al., 2014 [17]	Review article	N/A	United States	CDI molecular pathogenesis and novel therapeutics	The normal microbiome of the colon acts as an inhibitor for *C.* *difficile* colonization and certain bile salts can also inhibit *C.* *difficile* germination. Approximately 10% of C. *difficile* strains produce binary toxin which are clostridial iota-like toxins and have a higher rate of rCDI.
Kucharzik et al., 2023 [18]	Guideline	N/A	Europe	ECCO guidelines on CDI in IBD	IBD is known to be an independent risk factor for CDI, even in the absence of other risk factors. Screening for *C.* *difficile* is recommended during IBD flares. Suspected patients should be maintained under contact precautions until testing has confirmed the diagnosis. A two-step algorithm should be employed for diagnosis.
Berg et al., 2013 [19]	Review article	N/A	United States	CDI diagnosis in IBD	Patients with IBD are at higher risk of developing severe clinical symptoms and worse clinical outcomes. An independent risk factor is corticosteroids. *C. difficile* can occur in the small bowel in this patient population, especially in patients who have an ileoanal anastomosis (J-pouch).
Guery et al., 2020 [20]	Review article	N/A	Switzerland	Evaluate diagnostic modalities of CDI in the ICU	Clinical features and two-step algorithms are considered as a gold standard diagnostic test for C. diff in the ICU.
Lee et al., 2021 [21]	Retrospective cohort	23	Taiwan	Diagnosis of CDI in critically ill patients with diarrhea and ileus	In this ICU, 23 cases of CDI were included with six cases having ileus and 17 with diarrhea. The ileus group had a higher mortality rate, severe presentation, and shorter ICU stay.
Mileto et al., 2019 [22]	Review article	N/A	Australia	Overview of diagnostic testing for CDI	Current diagnostic options for C. *difficile* include CCNAs, EIAs, bacterial cultures, NAATs, and toxin detection tests.
Ong et al., 2017 [23]	Review article	N/A	United States	Overview of CDI diagnosis	Patients with CDI have diarrhea with at least three unformed stools in the last 24 hours or evidence of ileus or toxic megacolon with positive testing. Patients who have IBD are at a higher risk of developing CDI.
Mostafa and Abd el Hamid, 2022 [24]	Cross-sectional study	80	Egypt	Compare sensitivity and specificity of PCR in comparison to toxigenic culture for diagnosis of CDI in patients taking antibiotics	Out of 80 patients with AAD included in the study 12 (15%) were positive and 68 (85%) were negative for TC. Out of 80 patients with AAD included in the study, 32 (40%) were positive and 48 (60%) were negative for PCR.
Bouza et al., 2016 [25]	Review article	N/A	Spain	Diagnostic testing of CDI	TC is still the gold standard. Toxin EIAs have a short turnaround time but low sensitivity and high specificity and GDH EIA has a high sensitivity and low specificity. Newer PCR tests have high specificity and sensitivity and are faster but come with a cost.
Guery et al., 2019 [26]	Review article	N/A	Switzerland	Up-to-date data on diagnostic strategies for CDI	The ESCMID recommends a two-step approach that allows for a high sensitivity (NAAT or GDH assay) followed by a specific test for toxin if positive (EIA or CCNAs).
Usacheva et al, 2016 [27]	Review article	N/A	United States	CDI pathogenesis and molecular mechanisms of CDI-specific diagnostics	Currently, there is no gold standard test for diagnosis of CDI except for characteristic lesions visualized directly on colonoscopy. Patients with community-acquired diarrhea tested for *C.* *difficile* with PCR may falsely test positive due to colonization. New *C.* *difficile* strains with altered genomics and toxins may skew test results on currently available tests.
Srisajjakul et al., 2022 [28]	Review article	N/A	Thailand	Imaging characteristics associated with drug-induced bowel complications	Plain films may show large bowel dilation with a thumbprint appearance and CT scans commonly show colitis with wall thickening >1.5 cm. Imaging is used adjunctively when clinical diagnosis is in question.
Korman, 2015 [29]	Review article	N/A	Australia	Diagnosis of CDI in adults	Nontoxigenic *C.* *difficile *colonization is associated with a decreased risk of developing CDI. Many testing algorithms have been proposed such as the two-step approach, three-strep approach, and multistage algorithms.

Discussion

Risk Factors

Antimicrobials: There are many predisposing risk factors for CDI (Table 3), but the two main risk factors for CDI are exposure to antibiotics and *C. difficile* [1,3,11-16]. Antibiotic use is the strongest risk factor for the development of CDI, and the most common antibiotics include clindamycin, fluoroquinolones, and cephalosporins. Optimization of antimicrobial agents and antibiotic stewardship has been shown to reduce the incidence of CDI by up to 60% [4,30]. In the Netherlands, a three-year case-control study evaluated the association between duration and dosage of antibiotics. The third-generation cephalosporins had the highest odds of causing CDI, followed by carbapenems and second-generation cephalosporins (OR 5.3, 4.7, and 3.3, respectively) [31]. Those currently on antibiotics and within 30 days of completion had the greatest risk (OR 6.7-10.4) [11]. Interestingly, linezolid has conflicting data on CDI risk as some research has shown a theoretical inhibition of exotoxin production and reduction in *C. difficile* inhibition [32,33]. However, Baines et al.'s study [32] in the Maine Medical Center was primarily an experimental in vitro gut model while the study by Valerio et al. [33] lacked external validity as the patient population of interest were principally heart transplant recipients and had a small sample size (n = 91). On the other hand, a study by Janarthanan et al. found that patients who underwent hematopoietic stem cell transplant (HSCT) were more prone to developing CDI with linezolid [13]. Along with a depressed immune system due to multiple other comorbidities, these patients lose their protective gut microbiome from gastrointestinal inflammation [3,13].

**Table 3:** Risk factors for initial and recurrent Clostridioides difficile infections. CDI, *Clostridioides difficile* infection; PPI, proton pump inhibitor; IBD, inflammatory bowel disease; rCDI, recurrent* Clostridioides difficile *infection

Increased risk				Reduced risk
Initial CDI		rCDI		Pulsed dose (every 48 hours) in rCDI , hand hygiene, barrier precautions, infection control programs, antibiotic stewardship
Independent	Dependent	Dependent	Independent	
Recent gastro-intestinal surgery (particularly colectomy, ileo-anal pouch, and ileostomy), recent exposure to anti-neoplastic agents, IBD, previous hospitalization, advanced age (>60), greater comorbid conditions, solid and hematopoietic transplant	Antibiotics, PPI in cirrhosis, sharing a room with a CDI patient	Non-C. *difficile* antibiotics, sharing a room with a CDI patient	Advanced age (>65), poor health status, solid and hematopoietic transplant	

Nonantimicrobial risk factors: Proton pump inhibitors (PPIs) are common medications used in all clinical settings that have been associated with CDI. A meta-analysis of approximately 299,000 participants from 23 retrospective studies demonstrated a CDI incidence of 64.9% in PPI users [34]. The meta-analysis concluded with judicious PPI prescriptions. However, the study had limitations as the length of duration of PPIs was not defined. In addition, it incorporated the ‘trim and fill’ method to adjust the asymmetrical funnel plot which can lead to over- or under-estimation of true measures in this meta-analysis. Other retrospective studies or systematic/meta-analyses determined both PPIs and histamine receptor-2 blockers increase CDI. Although it is generally accepted by the United States Food and Drug Administration (FDA) that PPIs increase CDI, there is considerable controversy based on the currently available literature [1,3,4,12].

Reduced risk: A detailed list of factors that decrease the risk of CDI is listed in Table 3. Binders that are commonly used for bile sequestration such as cholestyramine and colestipol have been shown to decrease the risk of CDI [17]. Instead of these resins, vancomycin is highly efficacious and clinicians should be reminded to set a timing interval between oral vancomycin and bile resins. Currently, 4,000 mg of cholestyramine is given three to four times daily and two to three hours after oral vancomycin [11,35]. Apart from medications, asymptomatic colonization is thought to be immunoprotective. Approximately 40% of patients with community care-associated *C. difficile* do not have antibiotic exposure [11]. In 10% of healthy adults, up to 50% of institutionalized patients, and neonates become asymptomatic reservoirs and spread these bacteria throughout the healthcare system [36]. Carriers have immunoglobulin G (IgG) antitoxin A and B antibodies against* C. difficile*, thereby, inhibiting toxin production. It has been postulated that earlier colonization of asymptomatic *C. difficile* may lead to robust memory immunity until the later decades of life. As antitoxin A and B antibodies production weans with aging and apoptosis, this poses a risk factor for CDI in the elderly [1,37].

Special Risk Populations

Hypogammaglobulinemia: As mentioned, humoral immunity protects against toxicogenic colonization of *C. difficile*. Patients with solid organ transplant(s) (liver, kidney, heart, and lung), may benefit from passive immunization for *C. difficile*. These immunosuppressed receipts receiving prophylactic antibiotics post-transplant have a prevalence of 1.0% to 30% for CDI [3]. In addition, hypogammaglobulinemia is an independent risk factor for CDI and rCDI. In a prospective study, 235 patients underwent heart transplants, and 35 developed CDI. Of these 35 patients, immunoglobulin levels were determined to be low in six of the seven tested individuals [3,11,38]. Although routine IVIG administration is not recommended, it should be considered in patients with hypogammaglobulinemia who have other conferring comorbid conditions for CDI.

Surgical: In the surgical patient, there are multiple risk factors that both confound and modify the effect of CDI such as gastrointestinal surgery, emergent surgery, organ transplant, and nasogastric tube feeds [11]. Gastrointestinal surgeons influence CDI both directly (by surgical treatment) and indirectly (by inadvertently contributing to CDI by an unrelated surgery). There is evidence that enteral tube feeding in patients with anatomical or dynamic obstructions increases the risk of CDI. The transit time of stool is decreased and allows for the proliferation of toxins resulting in toxin proliferation [39]. According to the 2017 Infectious Disease Society Association (IDSA), a matched cohort study demonstrated enteral feeds increase the risk of CDI [40]. Therefore, it is best practice to discontinue nasogastric tube (NGT) early to reduce the possibility of cross-contamination from hospital instruments [1,3].

Inflammatory bowel disease (IBD): IBD harbors a pro-inflammatory state that causes physiological, anatomical, and immunological changes to the gastrointestinal tract [18]. IBD-specific populations present with community care-associated CDI. A national prevalence survey found CDI in ulcerative colitis (UC) to be 37 per 1,000, 11 per 1,000 in Crohn’s disease (CD), and four per 1,000 in general medical patients [41]. IBD patients suffer from acute flares leading to increased hospitalizations, immunosuppression with corticosteroids, and increased prescription of antimicrobials. Of all the aforementioned factors, corticosteroid administration has the greatest risk, with a threefold increase in CDI incidence [4]. A study in British Columbia determined that corticosteroids are an independent risk factor in IBD [19]. Interestingly, it is unclear what risk immunotherapy poses in this population. The complications of CDI are much higher in UC (9.5%) than with CD (7%) partly due to more extensive involvement of the colonic mucosa in UC [19]. Patients with colectomy and have ileo-anal pouch or ileostomy remain at an elevated risk of CDI as well. Symptoms such as increasing ostomy output, bleeding, changes in stool consistency and frequency, and systemic markers of inflammation should prompt evaluation of an infectious source. Healthcare professionals should have a low threshold to initiate therapy however should be aware of rising metronidazole resistance in this group of patients [3]. In terms of testing, IBD patients are more likely to have toxin-positive strains if there is one or more classic risk factors for CDI (antibiotic exposure, recent hospitalization, institutionalized, history of surgery) in comparison to toxin-negative strains (68% vs 31%) [14].

Intensive care unit (ICU): Patients who are directly admitted to the ICU are colonized with toxicogenic *C. difficile* strains. Approximately 15% of 5,300 admitted patients were confirmed to have CDI and this correlates to the increased incidence of community-acquired CDI [20]. Patients found to have CDI at the time of ICU admission were much more likely to have subsequent CDI in the future (p < 0.01) [42]. A retrospective study in Taiwan found that the diarrheal group had a longer length of ICU stay than the ileal group (28 vs 12 days, p < 0.01) [21]. A cohort study determined that the size of the room and the capacity of the room were related to horizontal transmission [43]. Therefore, hand hygiene is the cornerstone in the ICU to decrease the transmission of spores. In the ICU, there is an accumulation of co-morbid conditions, virulent organisms, and the use of broad-spectrum antibiotics that increase CDI risk.

Diagnosis of CDI

CDI diagnosis requires a clinical syndrome accompanied by a biochemical test for confirmation. CDI is defined as the presence of detectable toxicogenic *C. difficile* strain and clinical syndrome of acute diarrhea consistent with >3 unformed Bristol 5-7 stools in the last 24 hours without another explanation and prior exposure to antibiotics in the last two months. Each test must be accurate to diagnose the pathogen and timely to ensure rapid isolation for infection control and preventing progression. *C. difficile* colitis can be a challenge to diagnose as symptoms can overlap with other general diarrheal illnesses and detection of nontoxigenic strains of *C. difficile* which do not require treatment. To diagnose a CDI, patients must have acute diarrhea in addition to either toxigenic strain or *C. difficile* toxins in stool samples [22]. *C. difficile* tests include toxigenic culture (TC), glutamate dehydrogenase (GDH) detection assays, nucleic acid amplification tests (NAATs), cell cytotoxicity neutralization assays (CCNAs), and toxin detection tests (EIA) [37-39]. Patients who are at high risk for CDI are the ones who have received antibiotics in the last three months, hospitalized for more than three days, and are at least 65 years of age [3].

**Figure 2:**
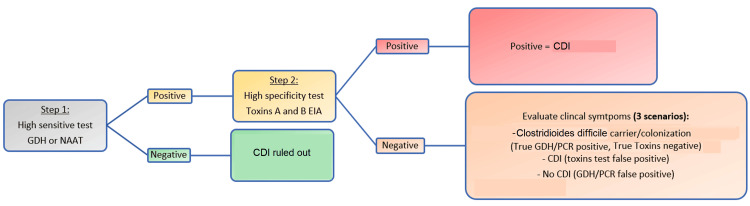
Two-step diagnostic approach for Clostridioides difficile GDH, glutamate dehydrogenase; EIA, enzyme immunoassay; CDI, Clostridioides difficile infection; PCR, polymerase chain reaction; NAAT, nucleic acid amplification test.

Diagnostic algorithms: No stand-alone test can distinguish between toxigenic/nontoxigenic strains and asymptomatic colonization (Table 4) [6]. Therefore, two-step or multi-step diagnostic algorithms have been used to improve the diagnosis of CDI (Figure 2). The two-step approach starts with a high sensitive test (GDH EIA or NAAT) followed by a high specificity confirmatory test (TcdA/TcdB EIAs) per the 2016 European Society of Clinical Microbiology and Infectious Diseases (ESCMID) (Table 5) [9,44,45].

**Table 4:** Detection of Clostridioides difficile in comparison to detection of toxins TC,toxigenic culture; GDH, glutamate dehydrogenase; EIA, enzyme immunoassay; PCR, polymerase chain reaction; NAAT, nucleic acid amplification test; TcdA, toxin A gene; TcdB, toxin B gene; CCNAs, cytotoxicity neutralization assays

Detection of *Clostridium difficile*	Detection of toxins A and/or B
TC (toxigenic *C.* *difficile* strain)	CCNAs: Detects TcdA and TcdB
GDH EIA (Toxigenic and nontoxigenic strains)	Toxin A B immunoassays (ELISA)
PCR-NAAT: Detects TcdA and TcdB genes (Toxigenic strains)	

**Table 5:** Clostridioides difficile test with their results, turnaround time, sensitivity/specificity, and important details. Sn, sensitivity; Sp, specificity; TC, toxigenic culture; GDH, glutamate dehydrogenase; EIA, enzyme immunoassay;NAAT, nucleic acid amplification test; CCNA, cytotoxicity neutralization assay; ELISA, enzyme-linked immunosorbent assay

Type of tests	Turnaround (h)	Sn/Sp	Details
TC	48-120 hours [6,7]	87-100%/94-100% [8]	This is a gold standard test. Isolates toxigenic strains of *C. difficile* from the stool culture or rectal swab.
GDH/EIA	<2 hours [7,15]	>90%/80-85% [8,15,44]	Considered the first test to order for screening [32]. Quicker and more sensitive than toxin EIAs. The test uses antibodies to detect the presence of GDH, a cell wall-associated enzyme that is present in both toxigenic and nontoxigenic strains. Therefore, cannot be used alone in the diagnosis of CDI.
NAAT	<4 hours [7]	100%/70% [15,24]	Detects nucleic acid sequences through amplifications of the genes that produce toxins A and B (TcdA and TcdB, respectively). Detects toxin genes instead of active toxin, it cannot differentiate between CDI and asymptomatic carriage [35]. NAAT can be done by PCR (polymerase chain reaction) or LAMP (loop-mediated isothermal amplification).
CCNAs	72-96 hours [22]	90-100%/98-99% [8]	This test works by inoculating a stool sample onto two sets of sensitive tissue culture cells, first set without *C. difficile* antitoxin and the second set with the antitoxin. Positive if cytopathic effect in the first set.
Toxin ELISA	<2 hours [7]	53-85%/91-98% [8]	This test uses antibodies to detect the presence of *C. difficile *toxins A/B. A negative toxin assay does not rule out toxigenic strains. Combining a high-sensitivity test (like GDH) with EIA can make up for the low sensitivity of this test.

**Figure 3:**
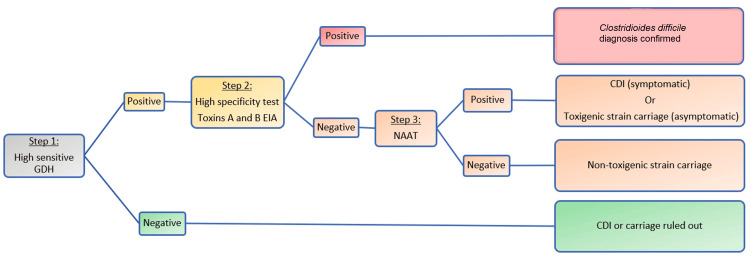
Multistep approach of the original two-step algorithm GDH, Glutamate dehydrogenase; EIA, enzyme immunoassay; CDI, *clostridioides difficile* infection; C. diff, *clostridioides difficile*; NAAT, nucleic acid amplification test.

Most algorithms start with a GDH test followed by toxin EIA. Some algorithms start with NAAT instead of GDH followed by toxin EIA, which is more expensive but has a higher diagnostic accuracy [25]. Economic studies showed that starting with GDH costs approximately $10 per algorithm vs $30 per algorithm when starting with PCR. Therefore, many small community hospitals and long-term care facilities perform GDH with toxin A/B EIA and subsequent NAAT for inconsistent results. Therefore, many small community hospitals and long-term care facilities perform GDH with toxin A/B EIA and subsequent NAAT for inconsistent results [6]. This is referred to as the multi-step approach (Figure 3), where the NAAT is used to differentiate if the positive GDH was due to a toxigenic strain (infection/colonization) or a nontoxigenic strain (colonization) [8].

**Figure 4:**
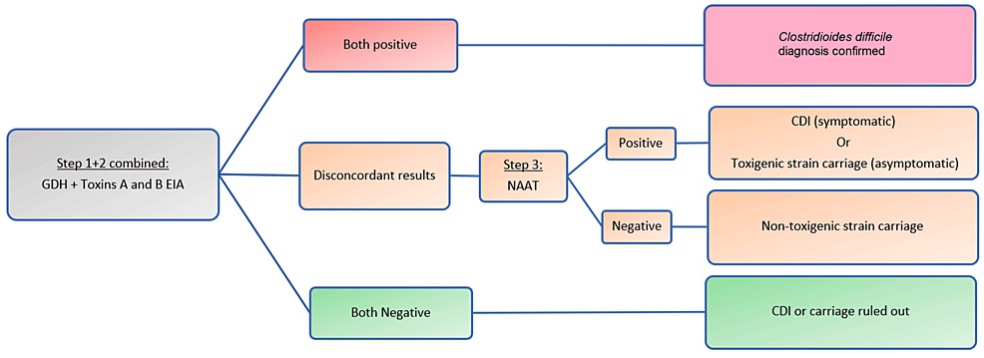
Modification of the multistep approach GDH, Glutamate dehydrogenase; EIA, enzyme immunoassay; CDI, *Clostridioides difficile* infection; NAAT, nucleic acid amplification test

Another modification of the multistep algorithm is to combine step 1 and step 2 by testing for GDH and toxins simultaneously, followed by NAAT in case of inconsistent results (Figure 4). The advantage of this modification is saving time by combining steps 1 and 2 (Table 6). The disadvantage is the cost of the toxins EIA test which is usually unnecessary if the GDH is negative. The previous three algorithms summarize the recommended testing by the ESCIMD Study Group for *C. difficile* (ESGCD) and ESCMID [9,26].

**Table 6:** Comparison of two-step, multistep, and modified multistep algorithms NAAT, nucleic acid amplification test; GDH, glutamate dehydrogenase; EIA: enzyme immunoassay

Algorithm	First test	Second test	Third test	Advantages/Disadvantages
2 step	NAAT	Toxin A/B EIA	N/A	Pros: Faster; Cons: More expensive
Multistep	GDH	Toxin A/B EIA	NAAT	Pros: Most cost-effective; Cons: Time-consuming, Multiple steps.
Modified multistep	GDH and Toxin A/B EIA		NAAT	Pros: Fast turnaround time; Cons: Unnecessary toxins test with GDH is negative

Who Should be Tested?

*C difficile* colonization has been reported to be 0-15% in healthy and 10-15% in hospitalized patients [8,27]. Therefore, screening symptomatic individuals to avoid false positives is crucial as laboratory tests alone cannot differentiate between an actual infection and asymptomatic colonization [8]. Fecal swabs cannot be used for toxin detection (inadequate sample) due to inadequate sample, instead, they can only be used for culture or NAAT [2,6].

Candidates for testing are those that are laxative-free for 48 hours, Bristol 5 or more >3 bowel movements in 24 hours, abdominal pain/cramps, with no other clear cause of diarrhea [8]. Patients with findings of colitis, severe ileus, or megacolon on imaging should be prioritized [6,28]. Additionally, early surgery consultation is recommended for those showing evidence of megacolon or ileus on imaging [4].

Retesting individuals within seven days of a previous negative test is not recommended. Repeated testing can increase healthcare costs and false-positive results [29]. The diagnostic yield of repeat testing is approximately 2%. Repeating testing to check for a cure is also not recommended, as greater than 60% of patients will remain positive after successful treatment [2,40].

Limitations

The inclusion and exclusion criteria limit this systematic review. This systematic review was governed by testing and diagnosis in specific populations only. The majority of the included reports in this systematic review were review articles and guidelines. In addition, there is significant heterogenicity in different studies that evaluated the risk factors and correlation of CDI. More randomized controlled and blinded studies need to be conducted in the future for specific risk factor attributes that may increase and/or decrease CDI prevalence and incidence.

## Conclusions

*C. difficile* is a major cause of healthcare-associated infections. There is an increased prevalence of this bacteria in the community from widespread contamination and transmission. The new emergence of the B1/NAP1/027 strain has caused widespread mortality and increased testing. The infection is a clinical syndrome that is defined by >3 unformed Bristol 5-7 bowel movements in the last 24 hours without another identifiable cause and positive stool testing. There are a wide variety of available diagnostic tests and the preferred tests are GDH antigen, toxin A/B EIA, and NAAT. These tests are accurate and make a timely diagnosis in the patient but we must be aware of the limitations of each test.

Toxin A/B EIA is not very sensitive while NAAT detects both toxigenic and non-toxigenic strains and does not differentiate between active disease or carrier state. The two-step approach, multi-step approach, and modified multistep approach are the different algorithms used for testing and vary due to cost and institution. Specific populations are more predisposed to *C difficile* colitis due to dysregulation in the immune system, medications, and the acuity of care. There is a need for further research in specific disease groups as studies have numerous variables that produce heterogeneity and poor external validity.
